# Identification and Validation of a DNA Damage Repair-Related Signature for Diffuse Large B-Cell Lymphoma

**DOI:** 10.1155/2022/2645090

**Published:** 2022-10-14

**Authors:** Yang Li, Xiyang Liu, Yu Chang, Bingjie Fan, Chenxing Shangguan, Huan Chen, Lei Zhang

**Affiliations:** Department of Oncology, The First Affiliated Hospital of Zhengzhou University, No. 1 Jianshe East Road, Zhengzhou 450000, China

## Abstract

**Background:**

Diffuse large B-cell lymphoma (DLBCL) is the most common subtype of non-Hodgkin's lymphoma in adults, whose prognostic scoring system remains to be improved. Dysfunction of DNA repair genes is closely associated with the development and prognosis of diffuse large B-cell lymphoma. The aim of this study was to establish and validate a DNA repair-related gene signature associated with the prognosis of DLBCL and to investigate the clinical predictive value of this signature.

**Methods:**

DLBCL cases were obtained from The Cancer Genome Atlas (TCGA) and Gene Expression Omnibus (GEO) databases. One hundred ninety-nine DNA repair-related gene sets were retrieved from the GeneCards database. The LASSO Cox regression was used to generate the DNA repair-related gene signature. Subsequently, the level of immune cell infiltration and the correlation between the gene signature and immune cells were analyzed using the CIBERSORT algorithm. Based on the Genomics of Drug Sensitivity in Cancer (GDSC) database, the relationship between the signature and drug sensitivity was analyzed, and together with the nomogram and gene set variation analysis (GSVA), the value of the signature for clinical application was evaluated.

**Results:**

A total of 14 DNA repair genes were screened out and included in the final risk model. Subgroup analysis of the training and validation cohorts showed that the risk model accurately predicted overall survival of DLBCL patients, with patients in the high-risk group having a worse prognosis than patients in the low-risk group. Subsequently, the risk score was confirmed as an independent prognostic factor by multivariate analysis. Furthermore, by CIBERSORT analysis, we discovered that immune cells, such as regulatory T cells (Tregs), activated memory CD4+ T cells, and gamma delta T cells showed significant differences between the high- and low-risk groups. In addition, we found some interesting associations of our signature with immune checkpoint genes (CD96, TGFBR1, and TIGIT). By analyzing drug sensitivity data in the GDSC database, we were able to identify potential therapeutics for DLBCL patients stratified according to our signature.

**Conclusions:**

Our study identified and validated a 14-DNA repair-related gene signature for stratification and prognostic prediction of DLBCL patients, which might guide clinical personalization of treatment.

## 1. Introduction

Diffuse large B-cell lymphoma (DLBCL), with an estimated 150,000 new cases per year worldwide, is the most common subtype of non-Hodgkin lymphoma among adults, accounting for 30%-40% of all cases [1]. Due to its heterogeneity in clinical manifestations, histomorphology, and molecular and genetic levels, approximately 40% of patients still cannot achieve long-term survival with standard treatment rituximab plus cyclophosphamide, doxorubicin, vincristine, and prednisone (R-CHOP) [2, 3]. The prognosis of patients with recurrence or refractory after first-line treatment is poor; only a minority of these patients can benefit from “salvage treatments” consisting of high-dose chemotherapy and autologous stem cell transplantation (ASCT) [2, 4]. Therefore, to improve prognosis, it is essential to stratify patients, according to risk level at the time of diagnosis and individualized treatment.

The International Prognostic Index (IPI) is a clinically validated tool for estimating the risk of death in patients with DLBCL that takes into account a variety of factors, including patient's age, performance status, number of extranodal sites of disease, hemoglobin level, lactate dehydrogenase level, and presence or absence of B symptoms [5]. However, since DLBCL is a genetic and molecular heterogeneity disease with a wide range of clinical presentations and outcomes, the IPI does not reflect the underlying molecular and biological mechanisms [6].

DNA damage can be a result of a variety of different factors, including exposure to ultraviolet light, radiation, and carcinogens. DNA repair or DNA damage response plays a crucial role in fixing DNA damage and maintaining genetic stability. In some types of cancer, the DNA repair machinery is defective, and cells are more likely to mutate, which allows the mutated gene products to exert their effects without restraint. And DLBCL is a kind of cancer that could arise when the DNA repair machinery malfunctions. In recent years, the role of DNA repair genes in tumorigenesis and prognosis of DLBCL has been extensively studied. For example, P53 is an important DNA damage response gene, which has been found mutated in about 20% of cases of DLBCL [7, 8]. Moreover, mutations of the TP53 tumor suppressor gene have been reported to be associated with poor survival in DLBCL [8, 9]. Another DNA damage response gene, ATM, which is one of the most frequently mutated genes in DLBCL, was correlated with a worse prognosis and proposed to be a potential new prognostic biomarker [10]. However, most studies have examined the role of DNA repair genes in DLBCL independently without considering the simultaneous changes of multiple genes. Therefore, to date, no comprehensive signature based on DNA repair genes has been constructed yet.

In the current study, we aimed to identify novel prognostic biomarkers for DLBCL that are associated with DNA repair genes. We established a prognosis-related signature based on 14 DNA repair genes through LASSO Cox regression analysis, which could predict the prognosis of DLBCL patients. In addition, we also found that the signature we constructed was highly correlated with immune cell infiltration and drug sensitivity. Finally, we created a predictive nomogram, as well as a ceRNA network with the DNA repair gene signature. In summary, we have constructed a novel DNA repair gene-related prognostic model, which might provide new insights into the prediction and treatment of DLBCL in clinics.

## 2. Materials and Methods

### 2.1. Data Collection

Series Matrix File data for GSE31312 were downloaded from the NCBI Gene Expression Omnibus (GEO) database (https://www.ncbi.nlm.nih.gov/geo/). The GSE31312 is based on the GPL570 Affymetrix Human Genome U133 Plus 2.0 Array, which consists of 470 DLBCL patients with complete expression profiles and a follow-up time of >0 days. A total of 47 original DLBCL mRNA expression data was obtained from The Cancer Genome Atlas (TCGA) database (https://portal.gdc.cancer.gov/) as a validation cohort to examine the signature we established.

### 2.2. Establishment and Validation of Prognostic Model

After DNA repair genes were selected, univariate Cox analysis was first performed to identify potential DNA repair genes significantly related to prognosis (*p* < 0.001). Then, these selected prognosis-related genes from the univariate Cox analysis were further included in the LASSO Cox regression analysis to build an optimal prognostic model using the R package “glmnet” [11]. Finally, a risk score formula was created by combining the relative expression of each gene and the gene expression levels weighted by the regression coefficients. Based on the risk score formula, patients were stratified into low-risk and high-risk groups using the median risk score as the cut-off value. Kaplan-Meier (K-M) survival curves and a log-rank test were used to assess the survival differences between the two groups. The time-dependent receiver operating characteristic (ROC) curves of 1-year, 2-year, and 3-year survival were performed to evaluate the validity of the model predictions. Survival analysis and the ROC curves were based on the survival and survivalROC packages [12]. The R package “rms” was used to generate the nomogram and calibration map.

### 2.3. Functional Annotation via Gene Ontology (GO) Analyses

The functional annotation of the prognostic genes was performed using the R package “ClusterProfiler” (version 3.14.3) to comprehensively investigate the functional relevance of these prognostically relevant genes [13]. The adjusted *p* < 0.05 and *q* < 0.05 were considered significant for GO analysis.

### 2.4. The Correlation Analysis between Prognostic Model and Drug Sensitivity

Based on the largest pharmacogenomic database, Genomics of Drug Sensitivity in Cancer (GDSC; https://www.cancerrxgene.org/), we used the R package “pRRophetic” to predict the chemosensitivity of each tumor sample by evaluating the half maximum inhibitory concentration (IC50) of each specific chemotherapeutic drug. The IC50 of each drug was estimated by ridge regression analysis, and the prediction accuracy was evaluated by 10-fold cross-validation with the GDSC training set. Default values were selected for all parameters, including “combat” to remove the batch effect, and duplicate gene expression was summarized as the mean [14].

### 2.5. Immune Infiltration Analysis

Normalized GSE31312 expression data were analyzed using the CIBERSORT algorithm (CIBERSORT R script v1.03) to estimate the relative proportion of 22 infiltrating immune cells [15], and Spearman correlation analysis was performed to evaluate the potential correlation between the gene signature and immune cells. *p* < 0.05 was considered statistically significant.

### 2.6. Gene Set Variation Analysis (GSVA)

Gene set variation analysis (GSVA) is a nonparametric, unsupervised method for assessing transcriptomic gene set enrichment. GSVA converts a gene-by-sample gene expression matrix into a gene set-by-sample pathway enrichment matrix by synthetically assessing gene sets of interest to determine the biological function of samples [16]. In this study, gene sets were downloaded from the Molecular Signatures Database (version 7.0), and the GSVA R package (version 1.34.0) was used to calculate the score of both low- and high-risk groups for individual samples.

### 2.7. ceRNA Network

Competitive endogenous RNAs (ceRNAs) have attracted considerable academic interest in recent years and represent a new model for regulating gene expression. Compared with the miRNA regulatory network, the ceRNA regulatory network is more sophisticated and complex involving more RNA molecules, including mRNA, pseudogenes, miRNA, lncRNA, etc. [17]. The NPInter database (version 4.0) is commonly used to query the relationship between lncRNA and miRNA. In this study, the NPInter database was used to predict lncRNA-miRNA interactions. In addition, FunRich software (version 3.1.3) was used to reversely predict the interactions between mRNA and miRNA. Then, the lncRNA-miRNA-mRNA network was constructed by combining lncRNA-miRNA interactions and mRNA-miRNA interactions and visualized using Cytoscape (version 3.7.1).

### 2.8. Motif Enrichment Analysis

Gene expression is regulated by transcription factors (TFs). Different transcription factors regulate different genes, which in turn cause cells to exhibit different states or types. To identify binding motifs of transcription factors enriched on 14 model genes, RcisTarget (version 1.6.0) was used to perform TF motif enrichment analysis to identify overrepresented TF binding motifs and to identify candidate transcription factors, where we applied a subset of 4.6 k motifs (rcistarget.hg19.motifdb.cisbpont.500 bp) to the gene-motif ranking database [18, 19].

### 2.9. Statistical Analysis

K-M survival curves and log-rank test were performed to compare the differences in overall survival (OS) between 2 different groups. The univariate and multivariate Cox proportional hazard models were used to calculate the hazard ratio (HR) of the variables and to determine independent prognostic factors of DLBCL. R software (version 3.6) was used for all statistical analyses. All statistical tests were two-sided, and *p* < 0.05 was considered statistically significant.

## 3. Results

### 3.1. Identification of Prognosis-Related DNA Repair Genes in DLBCL

**Figure 1:**
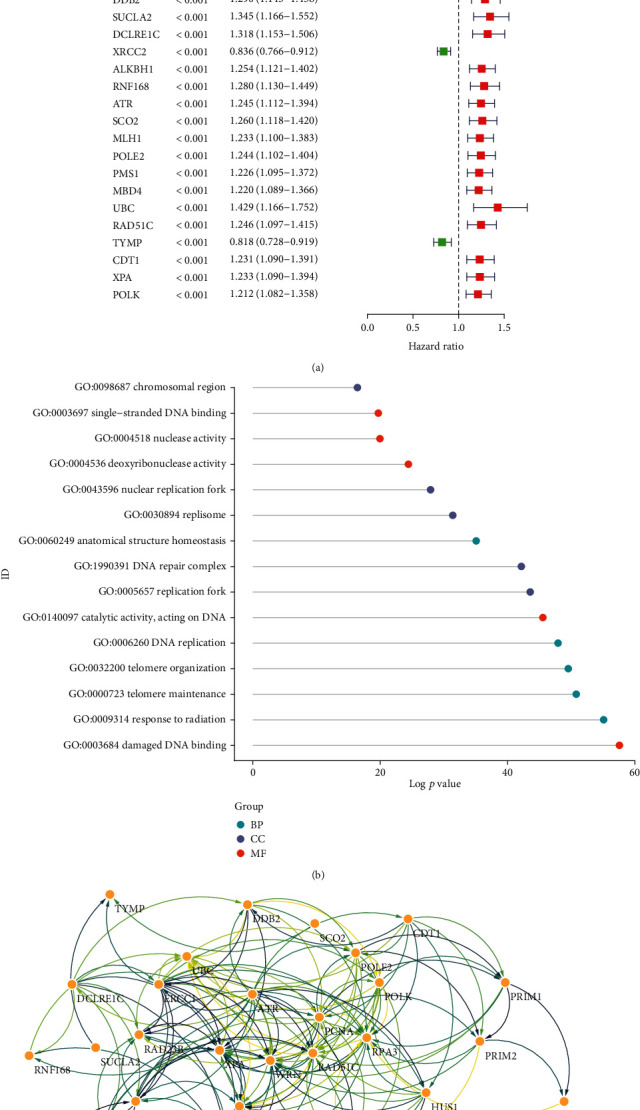
Identification of DNA repair genes associated with prognosis. (a) Forest plot shows the univariate Cox results of the 29 prognosis-related DNA repair genes (*p* < 0.001). (b) The GO enrichment analysis. BP: biological process; CC: cellular component; MF: molecular function. (c) PPI network indicating the interactions of the prognosis-related DNA repair genes.

We downloaded the original mRNA expression data (FPKM) of DLBCL from the GEO database (*n* = 470) and obtained a total of 199 DNA repair-related gene sets (relevance score > 20) from the GeneCards database (https://www.genecards.org/) in the meantime, of which 186 were included in the data from GEO. To further identify key genes in DNA repair-related gene sets, we first screened out 29 prognosis-related DNA repair genes (*p* < 0.001) for further investigation by collecting survival data from DLBCL patients using univariate Cox analysis (Figure 1(a), Table S1).

Twenty-nine prognosis-related DNA repair genes were enriched by GO analysis, and these genes were mainly associated with the binding of damaged DNA, response to radiation, telomere maintenance, catalytic activity, action on DNA, replication fork, DNA repair complex, and other pathways (Figure 1(b)). We also constructed a PPI network of these prognostic genes that was visualized using Cytoscape software (Figure 1(c)). The PPI network included 265 nodes and 1109 edges, of which RPA3 was identified as the most highly linked protein with the highest degrees (Table S2). The presence of a large number of links in this network indicates that these proteins interact frequently, suggesting that they may influence the prognosis of DLBCL patients.

### 3.2. Selection of the DNA Repair Genes and Construction of the Prognostic Gene Signature

**Figure 2:**
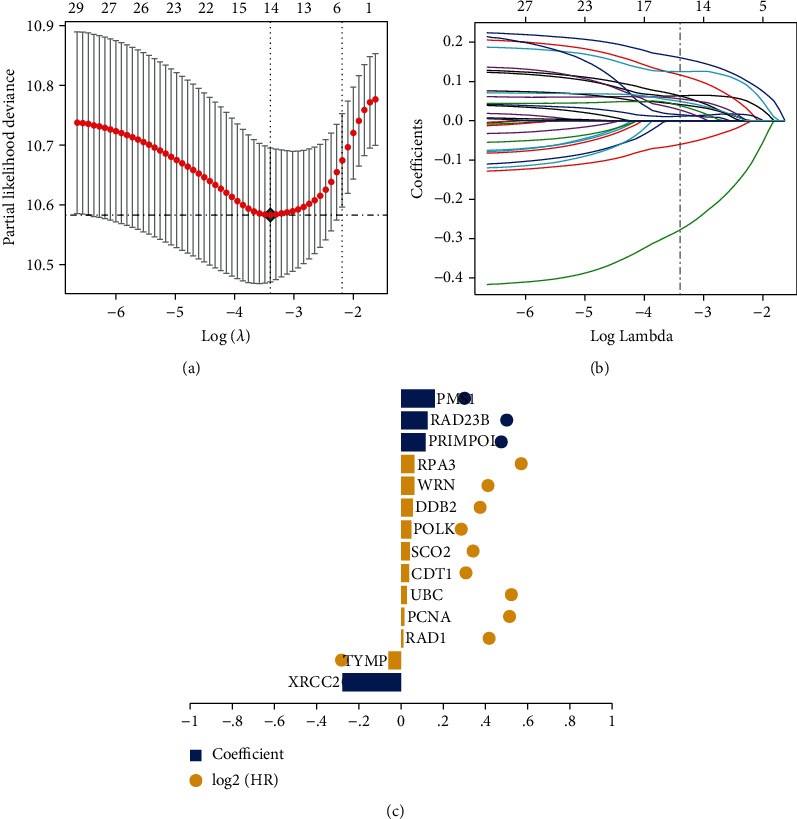
Construction of prognostic gene signature using LASSO regression analysis. (a) Tuning parameter selection by 10-fold cross-validation in the LASSO model. (b) LASSO coefficient profiles of the 29 candidate genes and the vertical dotted line at the optimal values. (c) The bar plot of the coefficients of fourteen genes in the prognostic model.

**Figure 3:**
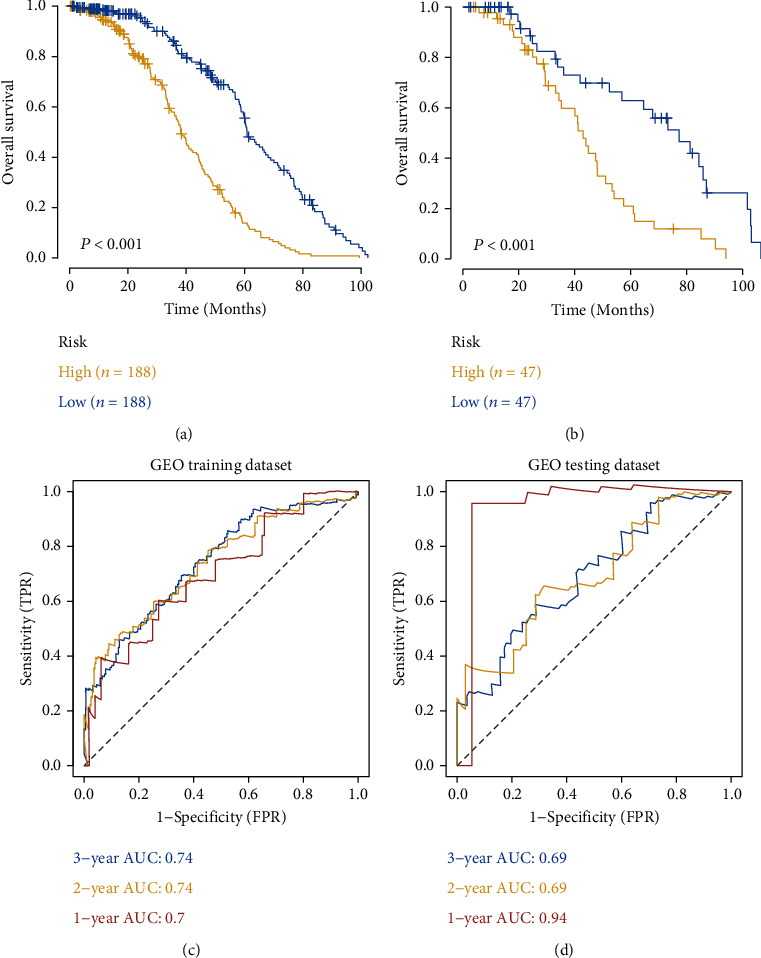
Survival analysis. (a, b) Kaplan-Meier curves of overall survival between high-risk and low-risk groups in both training and validation GEO-DLBCL cohorts (*p* < 0.001). (c, d) ROC curve of 1-, 2-, and 3-year overall survival in both training and validation GEO-DLBCL cohorts to assess the effectiveness of the risk model.

The 29 selected prognosis-related DNA repair genes were further included in the LASSO Cox regression analysis to eliminate the overfitting genes and construct an optimal prognosis-related signature for DLBCL. Patients in the GEO database were randomly divided into a training cohort and a validation cohort in a 4 : 1 ratio. We found that the prognostic model achieved the best performance when the 14 DNA repair genes were included (Figure 2(a)). Then, the optimal regression coefficients of these 14 genes were determined via LASSO Cox regression analysis (Figures 2(b) and 2(c)), Table S3) for subsequent modelling (risk score = XRCC2^∗^ (−0.27798) + TYMP^∗^ (−0.06018) + RAD1^∗^ (0.011479) + PCNA^∗^ (0.015372) + UBC^∗^ (0.027425) + CDT1^∗^ (0.03864) + sCO_2_^∗^ (0.041364) + POLK^∗^ (0.049191) + DDB2^∗^ (0.055706) + WRN^∗^ (0.06327) + RPA3^∗^ (0.063596) + PRIMPOL^∗^ (0.116672) + RAD23B^∗^ (0.12546) + PMS1^∗^ (0.160487)). Based on the risk score, patients were divided into a high-risk group and a low-risk group. K-M survival analysis showed that the OS of the high-risk group was significantly lower than that of the low-risk group in both training and validation cohorts (*p* < 0.001, Figures 3(a) and 3(b)). Moreover, the areas under the ROC curve (AUC) for 1 year, 2 years, and 3 years in the training cohort were 0.7, 0.74, and 0.74, respectively. And the AUC of the internal validation cohort was 0.94 at 1 year, 0.69 at 2 years, and 0.69 at 3 years, indicating that the model had good verification efficiency (Figures 3(c) and 3(d)).

### 3.3. External Validation

**Figure 4:**
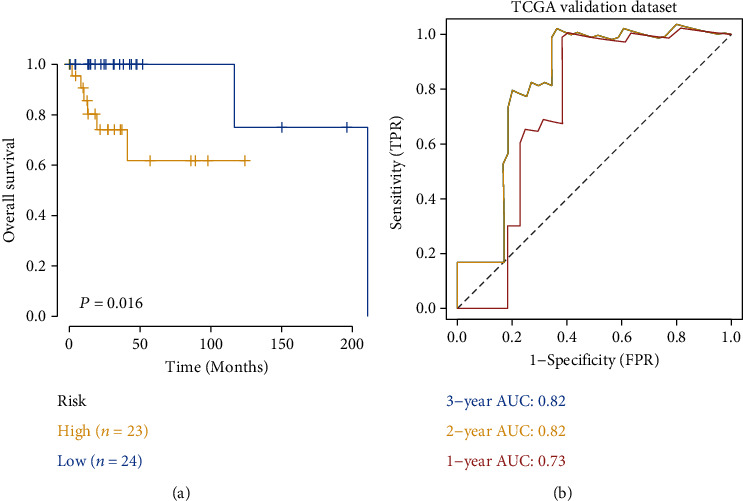
External validation of the prognostic signature using the TCGA-DLBCL cohort. Kaplan–Meier curves (a) and AUC curves (b) based on risk score in the TCGA-DLBCL cohort.

DLBCL patients from the TCGA database were downloaded to evaluate the robustness of the prognostic model we created (*n* = 47). Based on the risk score, patients were divided into a high-risk group (*n* = 23) and a low-risk group (*n* = 24). The difference in survival between the two groups was assessed by K-M analysis. We found that the results of the TCGA validation cohort were consistent with those of the entire GEO cohort, suggesting that high-risk patients were associated with an inferior prognosis (Figure 4(a)). To further determine the predictive accuracy of the external validation dataset, we constructed ROC curves, which showed strong efficacy of the model in predicting prognosis (AUC of 1, 2, and 3 years were 0.73, 0.82, and 0.82, respectively) (Figure 4(b)).

### 3.4. The Relationship between the Prognostic Signature and the Immune Microenvironment

**Figure 5:**
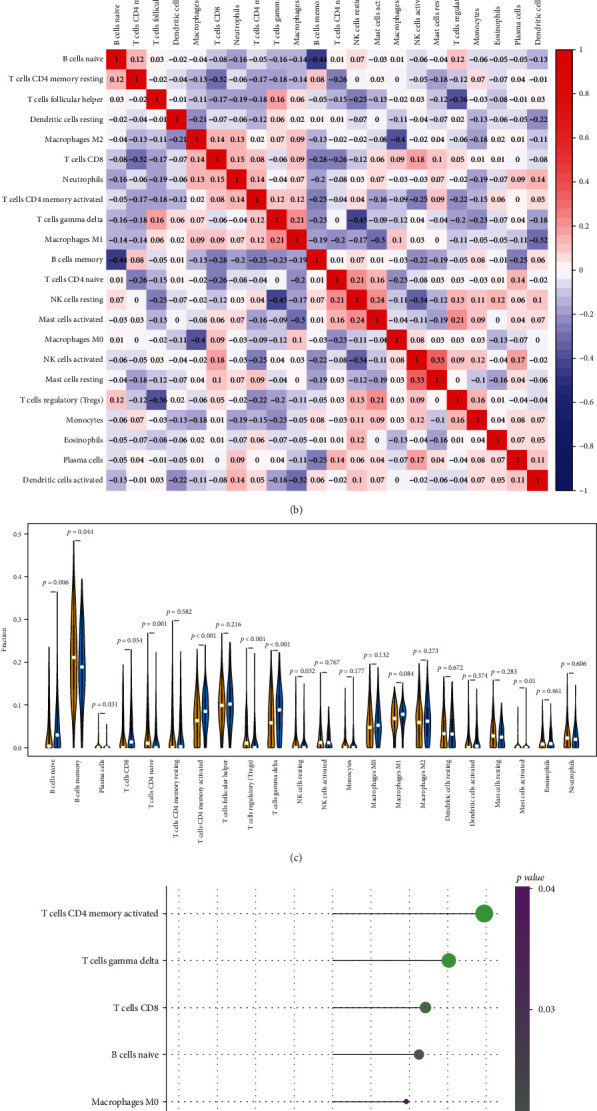
Immune cell distribution profiles. (a) Landscape of the composition of the 22 immune cells in each sample. (b) Correlation heat map of the 22 immune cells. The blue boxes indicate negative correlations, and the red boxes indicate positive correlations. (c) Violin plot comparing the proportion of immune cells between the high- and low-risk groups based on the risk score. The yellow bars represent the low-risk groups, and the blue bars represent the high-risk groups. (d) Correlation between risk score and infiltrating immune cells.

The tumor microenvironment is mainly composed of tumor-associated fibroblasts, immune cells, extracellular matrix, multiple growth factors, inflammatory factors, and specific physicochemical features (e.g., hypoxia and low pH), as well as the cancer cells themselves, which significantly affect the biological behavior, functions, and multidrug resistance of cancer cells. We further explored the potential molecular mechanism affecting the progression of diffuse large B-cell lymphoma by analyzing the relationship between the prognostic signature and tumor immune infiltration. The immune cell infiltration level of each sample was quantified using the CIBERSORT algorithm, and the correlation between 22 immune cells was further analyzed (Figures 5(a) and 5(b)), which showed that naive B cells were negatively correlated with memory B cells (*r* = −0.44) and gamma delta T cells were negatively related with resting NK cells (*r* = −0.43). Furthermore, we compared the differences in immune cell content between the low-risk group and the high-risk group. As shown, patients in the high-risk group had significantly fewer memory B cells (*p* = 0.044), naive CD4+ T cells (*p* = 0.001), and regulatory T cells (Tregs) (*p* < 0.001), while naive B cells (*p* = 0.006), activated memory CD4+ T cells (*p* < 0.001), and gamma delta T cells (*p* < 0.001) were significantly higher.

**Figure 6:**
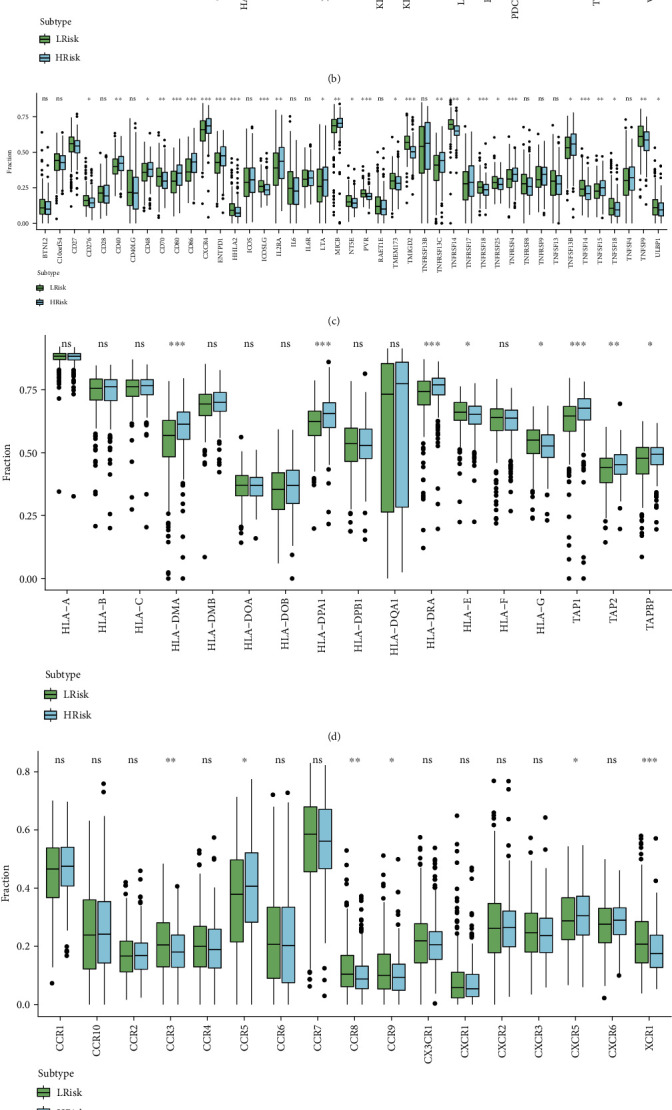
Boxplots showing the proportion of immune-related chemokines (a), immune inhibitors (b), immune stimulators (c), MHC (d), and immune receptors (e) in the high- and low-risk groups. *p* values: ^∗^ < 0.05, ^∗∗^ < 0.01, ^∗∗∗^ < 0.001. (f) Correlation between risk score of the signature and immunoregulatory genes. The area of the sector represents the magnitude of the correlation, with orange representing negative correlation and blue representing positive correlation.

The relationship between the signature and immunoregulatory genes was further analyzed. The differences in the expression of genes such as immune-related chemokines, immune inhibitors, immune stimulators, major histocompatibility complex (MHC), and immune receptors between high- and low-risk groups are shown in Figures 6(a)–6(e). We also examined the correlation between risk score and immunoinhibitory genes. The correlation heat map showed that several genes such as CD96, TGFBR1, and TIGIT were strongly positively correlated with the risk score (Figure 6(f)).

### 3.5. Activation of Signaling Pathways Related to the Signature

**Figure 7:**
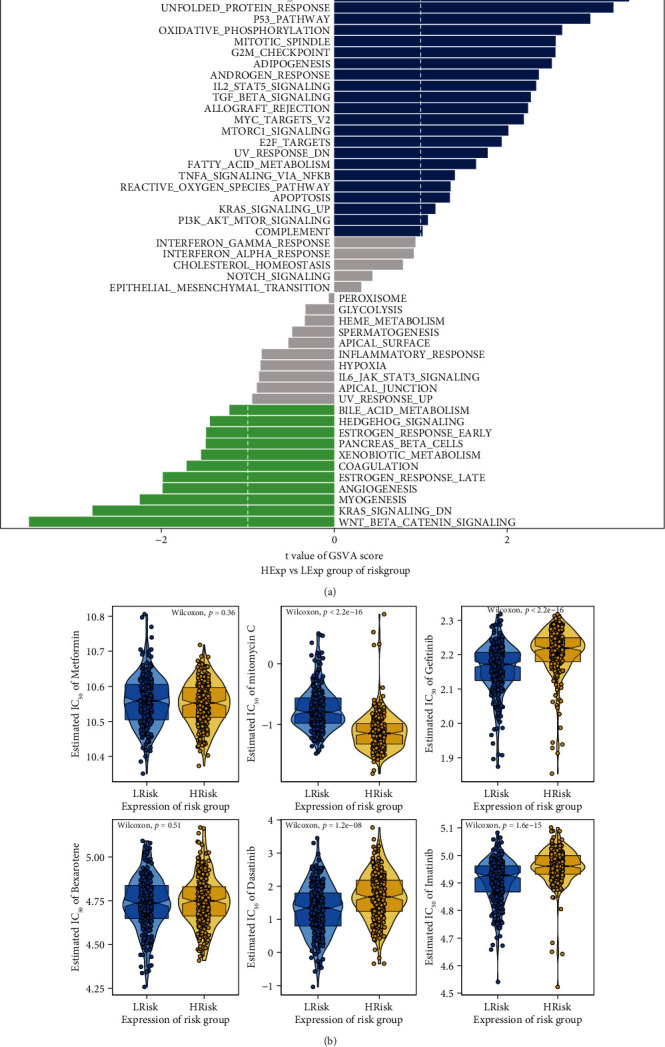
GSVA analysis and drug sensitivity. (a) Differences in signaling pathways enriched between the high-risk and low-risk groups. Blue bars indicate activated signaling pathways in the high-risk group, and green bars indicate activated signaling pathways in the low-risk group. (b) Comparison of IC50 of chemotherapeutic agents and targeted drugs between the high-risk and low-risk groups.

GSVA was used to identify the specific signaling pathways involved in the high- and low-risk groups to explore the potential molecular mechanisms by which signature influences tumor progression. The GSVA results showed that the high-risk group was enriched in the signaling pathways MYC_TARGETS, DNA_REPAIR, P53_PATHWAY, and OXIDATIVE_PHOSPHORYLATION, which are associated with DNA damage and DNA repair, and the abnormality of these pathways played a crucial role in tumorigenesis. Meanwhile, signaling pathways such as WNT_BETA_CATENIN_SIGNALING, KRAS_SIGNALING_DN, and MYOGENESIS were enriched in the low-risk group (Figure 7(a)). All these showed that the alterations of these signaling pathways affected the prognosis of patients in the high- and low-risk groups, indicating that the prognostic model we constructed is representative.

### 3.6. The Relationship between Signature and Drug Sensitivity

Immunochemotherapy, as one of the common treatments for DLBCL, is typically used in combination with common targeted therapies such as CD20 antibodies and tyrosine kinase inhibitors. Sensitivity to immunochemotherapy is also an important prognostic factor for DLBCL. Chemotherapy in combination with immunotherapy (e.g., rituximab) has been shown to be effective in the treatment of DLBCL. Based on the drug sensitivity data from the GDSC database, we further investigated the correlation between the signature and sensitivity to common chemotherapeutic agents and targeted drugs. As shown in Figure 7(b), the high-risk group was more sensitive to mitomycin C, while the IC50 values of gefitinib, dasatinib, and imatinib were significantly higher in the high-risk group than in the low-risk group, suggesting that DLBCL patients in the high-risk group were more resistant to these molecularly targeted drugs; i.e., the high-risk group was less sensitive to gefitinib, dasatinib, and imatinib. Our signature correlated with sensitivity to multiple drugs, which may indicate that the signature has potential predictive properties for sensitivity to chemotherapy. Overall, patients in the high-risk group may benefit more from chemotherapeutic agents than from these tyrosine kinase inhibitors (TKIs).

### 3.7. Incidence Risk and Independent Prognostic Analysis

**Figure 8:**
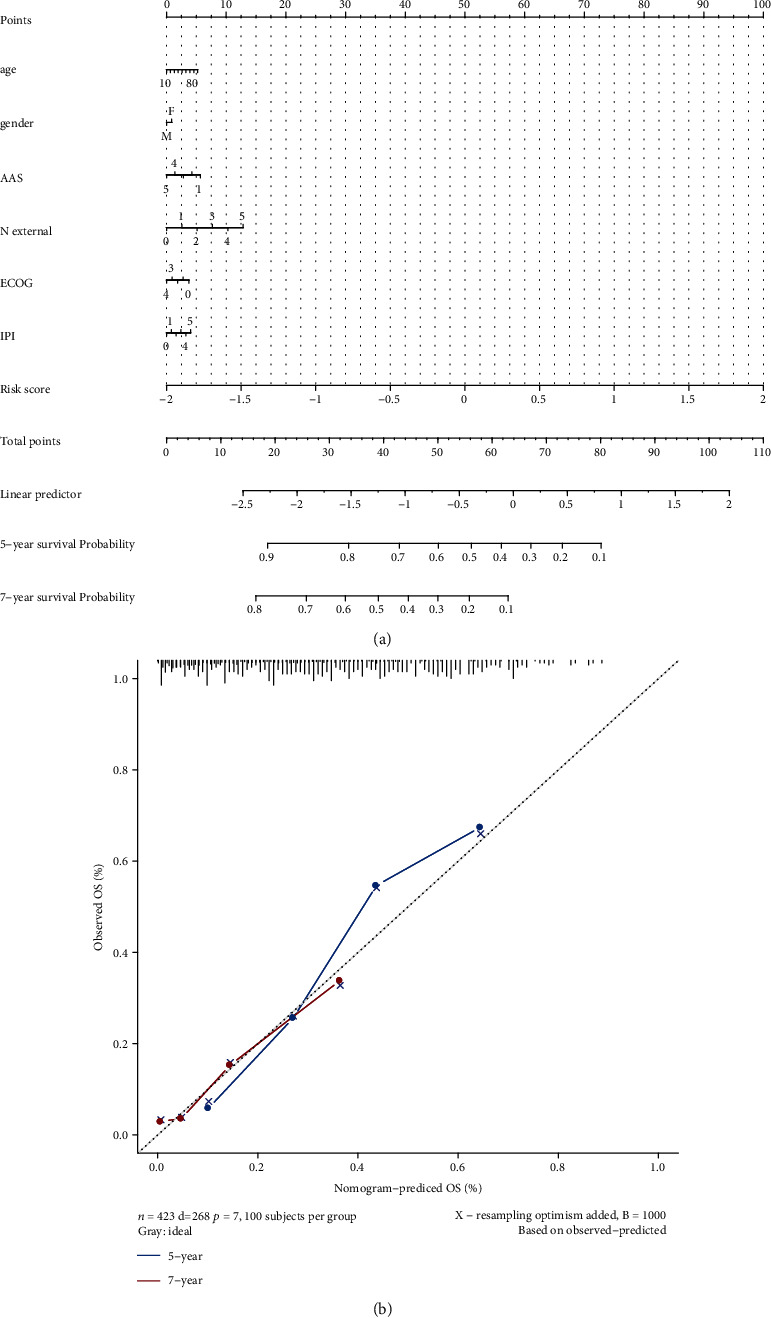
Predictive nomogram. (a) Nomogram predicting 5-year and 7-year survival probabilities in DLBCL patients. (b) Calibration curves of observed and predicted OS, to verify the accuracy of the nomogram.

**Figure 9:**
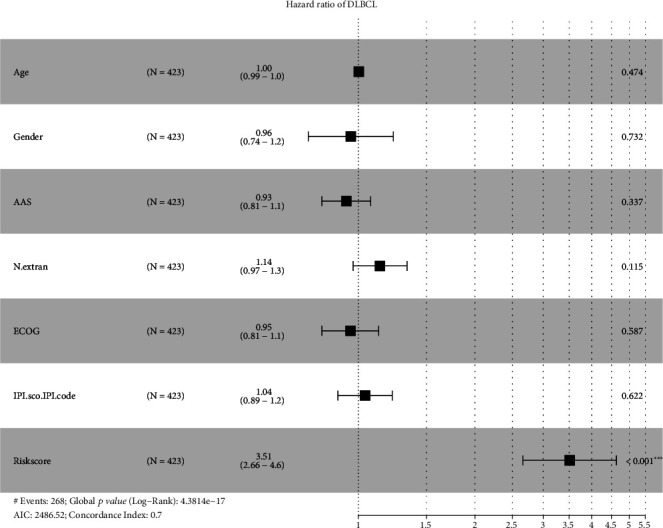
Forest plot of multivariable Cox analyses of the risk score and clinical characteristics. *p* value, ^∗∗∗^ <0.001.

To demonstrate the role of the signature in assessing the incidence risk and prognosis of DLBCL, we constructed a predictive nomogram based on age, gender, Ann Arbor staging (AAS), N extranodal, ECOG, IPI, and the risk score derived from our signature. By converting the regression coefficient of each variable from multivariate logistic regression analysis into a 0- to 100-point scale, we created the alignment diagram to predict the 5- and 7-year survival probability of DLBCL patients. Among all variables, the risk score contributed significantly to the nomogram prediction model, and the risk score was assigned 100 points in our nomogram (Figure 8(a)). The predictive accuracy of our nomogram was measured using calibration curves with 1000 bootstrap samples to reduce overfit bias by comparing the concordance between the OS predicted by the nomogram and the OS observed, underpinning its suitability for predicting survival in DLBCL (Figure 8(b)). In addition, we performed multivariate Cox regression analysis and confirmed that the risk score was an independent prognostic factor for DLBCL patients (*p* < 0.001) (Figure 9).

### 3.8. ceRNA Network

**Figure 10:**
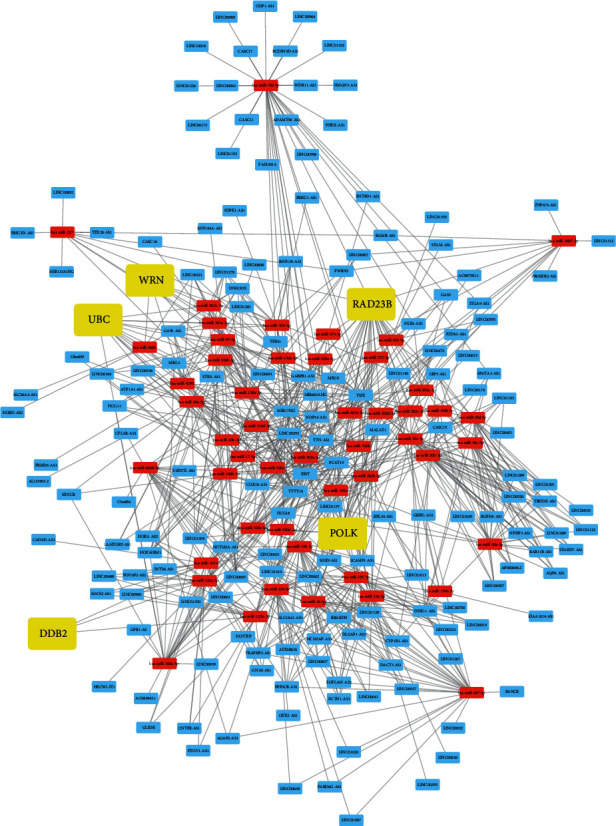
ceRNA network based on model genes. Yellow, blue, and red represent mRNAs, lncRNAs, and miRNAs, respectively.

First, FunRich was used for reverse prediction of 14 model genes, and 50 miRNAs were predicted that formed 69 mRNA-miRNA pairs. Then, reverse prediction of lncRNAs was performed using NPInter based on the 50 miRNAs, resulting in a total of 10,248 mRNA-miRNA-lncRNA pairs. To further screen the most important lncRNAs, we used maximum information coefficients (MIC) to calculate the correlation between lncRNAs and model genes and screened out 1785 lncRNAs (cor > 0.2). These 1785 lncRNAs were crossed with 10,248 mRNA-miRNA-lncRNA pairs to finally obtain 948 mRNA-miRNA-lncRNA pairs and construct a comprehensive ceRNA network associated with model genes (Figure 10).

### 3.9. Motif Enrichment Analysis

**Figure 11:**
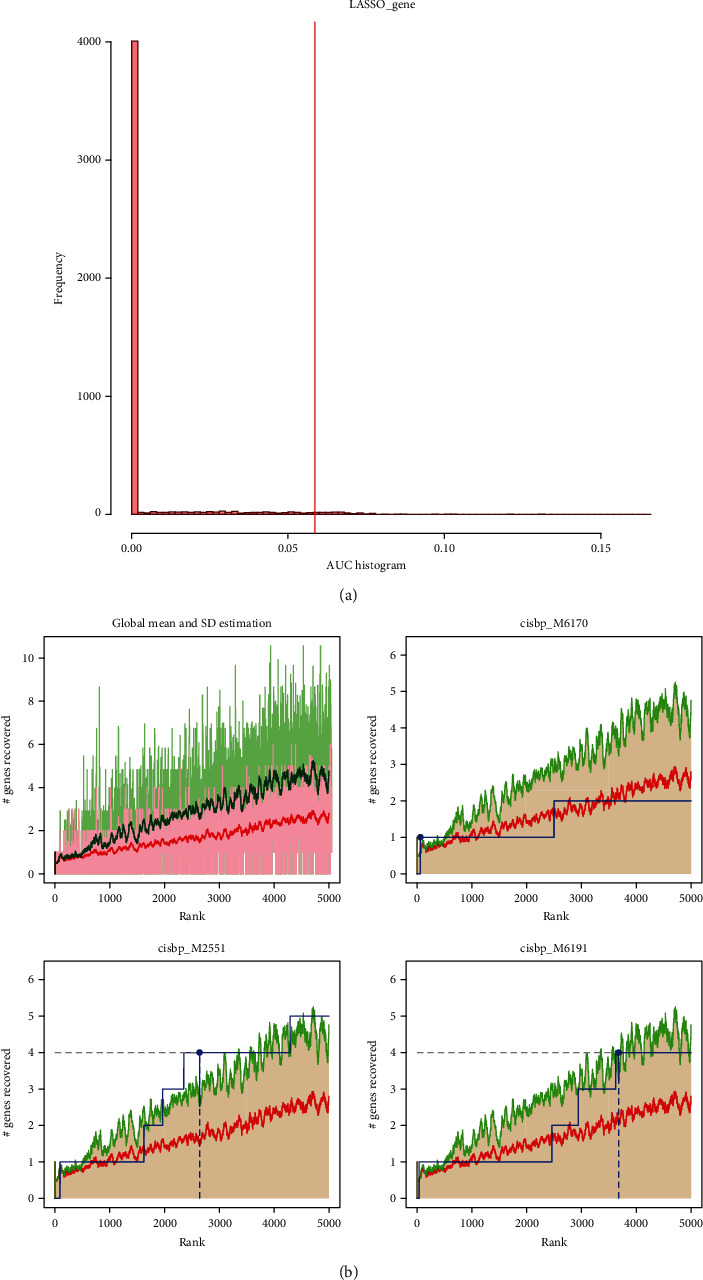
Motif enrichment analysis. (a) AUC histogram estimating the overrepresentation of each motif on our model genes. (b) Selection of the best enriched genes for each motif. The red line is the mean of the recovery curve for each motif, the green line is the mean + standard deviation, and the blue line is the recovery curve for the current motif. The maximum distance point (mean + sd) between the current motif and the green curve is the selected maximum enrichment level.

We used 14 model genes for motif enrichment analysis and found that they were regulated by multiple transcription factors. After a series of analyses to calculate enrichment using a recovery-based method, selecting significant motifs and motif TF annotation, and identifying the genes with the best enrichment for each motif, the transcription factor NRF1 emerged as the most important regulator in our gene set, MOT annotated as cisbp__M5688, in which a total of five model genes were enriched in this motif with a normalized enrichment score (NES) of 9.75. Here, we show all enriched motifs and the corresponding transcription factors of the model genes (Figures 11(a) and 11(b), Figure S1).

## 4. Discussion

DLBCL is the most common subtype of adult non-Hodgkin's lymphoma, with a 40% recurrence rate in the era of rituximab [20, 21]. To improve the prognosis of patients with DLBCL, it is critical to identify novel prognostic factors that can predict disease progression. Traditional prognostic stratification schemes such as the IPI and cell origin classification (COO) can be used to classify patients into clinical risk groups, which in turn helps in clinical decision making. However, these classification schemes have several limitations, and a consensus is emerging that DLBCL with the same clinical features, but different molecular and genetic characteristics, may represent a distinct entity and respond differently to specific therapy. Therefore, a more refined and accurate classification system is needed. In this study, a molecular classification for DLBCL was established to improve the prognostic stratification of patients with DLBCL. In addition, the important role of DNA repair genes in the development and progression of DLBCL was identified, which can be used as a prognostic factor.

Recent advances in molecular and cytogenetic techniques have allowed refinement of the classification of DLBCL. Several distinct genetic abnormalities, including somatic mutations in genes such as MYD88, CD79B, and MYC, have been identified in DLBCL, and single or combined mutations have been shown to have prognostic value in DLBCL subtypes [22–24]. Bioinformatics has been the forefront in advancing the field of cancer research and has been applied to many different areas, including the prognosis of diffuse large B-cell lymphoma. For example, TME immune cell signatures [21] and lncRNA signatures [25] have been identified for subtype classification and prognosis prediction of DLBCL patients. In addition, some integrated models such as the model incorporating pharmacogenomic gene signatures and clinical information as well as the IPI-based immunoprediction model have been validated to have better predictive power for DLBCL patients [26]. However, there is no study yet to declare whether there is a DNA repair-related prognostic signature capable of predicting the outcome of DLBCL.

DNA repair or DNA damage response (DDR) is a cellular program that detects DNA damage and triggers DNA repair to protect genetic material from damage. In a subset of DLBCL, mutations of genes involved in DNA damage response and DNA repair genes are identified, and defects of some DNA repair factors are associated with patient prognosis [27–29]. The aim of this study was to evaluate the prognostic value of DNA repair genes that play important roles in DLBCL and to develop a prognostic signature that can stratify DLBCL patients. First, we identified twenty-nine DNA repair genes that significantly correlate with overall survival in DLBCL patients. We then subjected them to LASSO Cox regression analysis to generate a signature of 14 DNA repair-related genes. Patients were divided into low-risk and high-risk groups according to the prognostic risk score formula. K-M analysis estimated survival probabilities and showed that the prognostic model based on these 14 DNA repair genes, including XRCC2, TYMP, RAD1, PCNA, UBC, CDT1, sCO_2_, POLK, DDB2, WRN, RPA3, PRIMPOL, RAD23B, and PMS1, was useful and robust in predicting survival in both our training and validation GEO-DLBCL cohorts as well as the external validation TCGA-DLBCL cohort. In addition, based on the risk score and other independent factors, we constructed a predictive nomogram to predict the prognosis of DLBCL patients to estimate their survival probability and identify high-risk patients. Among all variables, risk score significantly contributed to survival prediction. Moreover, the risk score based on the prognostic model proved to be a useful independent prognostic factor by multivariate analysis. Furthermore, we constructed the DNA repair-associated ceRNA network based on the hypothesis that RNAs interact by competing for a limited pool of microRNAs to reveal a novel mechanism of interaction between RNAs and probably provide insights for further studies on potential lncRNA biomarkers in DLBCL [17]. Finally, motif enrichment analysis was performed to identify the mechanisms controlling the transcription of these DNA repair genes. Interestingly, the transcription factor NRF1 was found to be the major regulator in our gene set. NRF1, a transcription factor, has been shown to be an important component of the DNA repair network in response to UVB-induced DNA damage, and NRF1 can also regulate circRNAs to promote lymphoma progression [30, 31]. All these suggest that the DNA repair gene signature is a robust prognostic stratification system that may serve as a novel prognostic indicator for DLBCL.

X-ray Repair Cross-Complementing 2 (XRCC2) is one of many proteins that regulate the repair of DNA double-strand breaks (DSBs) by homologous recombination [32]. Studies on genetic variations in XRCC2 (rs3218536, Arg188His) have revealed some evidence of association with breast cancer risk and poor survival [33–35]. Thymidine phosphorylase (TYMP) is a catabolic enzyme in thymidine metabolism that has been attributed a dual role in promoting angiogenesis and inhibiting apoptosis in many tumors [36, 37]. In DLBCL, TYMP has been shown to correlate with the development of a nongerminal center origin and a worse outcome [38]. It has been previously demonstrated that RAD1 can form a heterotrimeric complex (9-1-1 complex) that is activated to arrest cell cycle progression in response to DNA damage or incomplete DNA replication [39]. In ovarian cancer, RAD1 acts as a tumor suppressor in a BRCA-like manner [40]. Proliferating Cell Nuclear Antigen (PCNA) is a protein known to play a key role in DNA replication and repair in non-Hodgkin's lymphoma. It interacts with many different proteins involved in replication, repair, and tumorigenesis and is an important prognostic factor in non-Hodgkin's lymphoma [41–43]. Ubiquitin C (UBC) attaches to abnormal proteins to mark them for destruction by the degradation process known as ubiquitination. UBC has a variety of functions, a major one of which is the regulation of DNA repair [44]. Remarkably, upregulation of UBC has also been found in many human cancer specimens [45, 46]; however, the relationship between UBC and DLBCL needs further research. The checkpoint protein chromatin licensing and DNA replication factor 1 (CDT1) is a key regulator of DNA replication licensing and DNA damage repair [47]. Deregulation of CDT1 has been shown to trigger a DNA damage response that contributes to genomic instability, and CDT1 has been reported to function as an oncogene in various human cancers [48–50]. However, the relationship between CDT1 and DLBCL is still unknown. sCO_2_ is essential for the proper assembly of cytochrome c oxidase (COX), the terminal component of the mitochondrial respiratory chain [51]. As a transcriptional target of p53, sCO_2_ plays an important role in oxidative phosphorylation [52]. To date, the effect of sCO_2_ in different cancer types remains controversial. Low sCO_2_ expression has been associated with significantly worse prognosis in patients with breast cancer, while high sCO_2_ expression predicted worse survival in human lung adenocarcinoma [53, 54]. DNA polymerase kappa (POLK) is a member of the POLK family of DNA polymerases specifically involved in DNA repair. Dysregulation of POLK can lead to genetic instability in human cells, which is associated with some cancers. However, the role of POLK in DLBCL has not been adequately evaluated [55–57]. DNA damage is recognized by specific proteins that can initiate a signal transduction cascade. One of these proteins is damage-specific DNA-binding protein 2 (DDB2), which plays an important role in the initial steps of nuclear excision repair (NER) [58]. DDB2 appears to have a dual function in cancer, sometimes with tumor suppressor properties and sometimes as an oncogene [59–61]. It has been reported that the expression of WRN is related to the expression of Myc oncoprotein, which has been implicated in the pathogenesis of DLBCL [62, 63]. Replication protein A3 (RPA3) is part of the heterotrimeric replication protein A complex, which plays an essential role in DNA replication and DNA repair by binding to single-stranded DNA and damaged DNA on chromatin. RPA3 expression has been shown to correlate with poor prognosis and radioresistance in various cancers [64–66]. PRIMPOL is thought to contribute to cellular tolerance to DNA damage by facilitating damage bypass [67]. Further studies have shown that loss of PRIMPOL leads to increased cellular sensitivity to DNA cross-linking agents such as mitomycin C, suggesting that repriming of PRIMPOL may represent a novel target for improving the sensitivity of cancer cells to DNA-damaging chemotherapies [68, 69]. Rad23 homolog B plays a critical role in nucleotide excision repair (NER) in humans [70]. The study demonstrated that depletion of RAD23B abrogated the accumulation of ubiquitinated p53 and suppressed apoptosis after mitomycin C (MMC) exposure [71]. PMS1 protein homolog 1 (PMS1) is required for DNA mismatch repair, which is also involved in the repair of DNA damage caused by cisplatin, alkylating agents, and oxidation [72, 73]. Loss of function of PMS1 leads to microsatellite instability and colorectal cancer [74]. The role of this protein in DLBCL has not yet been established.

Additionally, to further explore the immune infiltration-related mechanism affecting DLBCL progression, the estimated proportions of 22 tumor-infiltrating immune cell types in DLBCL were evaluated by CIBERSORT analysis. It was found that memory B cells, naive CD4+ T cells and regulatory T cells (Tregs), naive B cells, activated memory CD4+ T cells, and gamma delta T cells showed significant differences between high- and low-risk groups. Recent studies suggest that the composition of the TME is essential for the pathogenesis of lymphoma [21, 75]. Regulatory T cells (Tregs) are important modulators of the interaction between lymphoma cells and the host microenvironment [76]. In DLBCL, intratumoral FOXP3+ Tregs are reported to be associated with superior prognosis, especially in the GCB type, but with poor prognosis in the non-GCB type [77, 78]. According to our results, patients in the high-risk group had significantly fewer regulatory T cells (Tregs), implying that Tregs may serve as suppressors. Gamma delta T cells (*γδ* T cells), as a bridge between innate and adaptive responses, can exhibit dual antitumor and protumor activity in cancer [79]. Although the *γδ* T cells make up only a minor proportion in TME, they have been demonstrated to be an important component of tumor-infiltrated lymphocytes in many types of cancer [80–82]. A previous study has shown a significant abundance of *γδ* T cells in DLBCL patients [83], but the role of *γδ* T cells in DLBCL is still unknown. In our study, we found that *γδ* T cells were positively correlated with the high-risk score, which may define the protumor role in DLBCL, but we need more evidence to further validate the function of *γδ* T cells in DLBCL. Activated memory CD4+ T cells are an important infiltrate in the microenvironment of DLBCL [84]. Tumors from DLBCL patients were enriched in CD4+ T memory cells that displayed high coexpression of TIGIT and PD-1, which may be potential targets for novel therapeutic intervention in DLBCL [85]. Immune checkpoint inhibition or blockade is a cancer treatment strategy that harnesses the power of the immune system to attack cancer cells. It has had a profound impact on the prognosis of many cancers and has recently been approved as an important treatment option for patients with DLBCL. Therefore, in our model, we investigated the relationship between risk score and immunoregulatory genes, especially genes related to immune checkpoints. We found that the expression of CD96, TGFBR1, and TIGIT was significantly increased in high-risk patients. Taken together, our analysis of immune infiltration suggests that the strong expression of immune inhibitors and immunosuppressive TME surrounding the tumor contribute to the poor prognosis of the high-risk group. Furthermore, we hypothesize that high-risk patients may respond more effectively to therapy with TIGIT, CD96, and TGFBR1 checkpoint inhibitors which provides us with additional clues for immunotherapy candidates.

Our GSVA analysis uncovered distinct molecular mechanisms between the low- and high-risk groups, with several DNA repair-related gene sets or signaling pathways, including the unfolded protein response pathway, the P53 pathway, and MYC targets, being most enriched in the high-risk group, whereas the Wnt/*β*-catenin and KRAS pathways were enriched in the low-risk group. MYC is thought to be associated with the development of multiple cancers, including DLBCL [86, 87]. And dysregulation of MYC can directly impair DNA repair and induce genomic instability [88, 89]. The P53 protein is an important tumor suppressor that plays a role in numerous cellular processes, including cell cycle arrest, apoptosis, and DNA repair. Conversely, loss of p53 function leads to loss of these processes, resulting in mutations and cancer development [90]. Unfolded protein response (UPR) is a conserved signaling pathway that responds to protein misfolding and the accumulation of unfolded proteins in cells [91]. When cells are under stress, both the UPR and the DNA damage response are activated, allowing cells to repair damage [92, 93]. Therefore, we hypothesize that the high-risk group may have a higher degree of malignancy, and thus, more damage occurs, leading to excessive activation of DNA repair signaling pathways. While the low-risk group showed a positive correlation with Wnt/*β*-catenin [94] and KRAS signaling [95], both are drivers or participants in various cancers, and their crosstalk between the DNA damage response has been validated, which may provide combination strategies for the treatment of cancer to overcome the dysregulation and deficits in DNA repair [96, 97]. In summary, these GSVA results describe the different molecules and mechanisms involved in the progress of low-risk and high-risk DLBCL, which provide a basis for future targeted therapeutic studies.

Subsequently, we performed a multiomics analysis to investigate the clinical utility of the prognostic model. First, analysis of drug sensitivity data from the GDSC database showed that the high-risk group was more sensitive to mitomycin C, whereas gefitinib, dasatinib, and imatinib were more effective in the low-risk group. Dasatinib is a tyrosine kinase inhibitor that can inhibit Src family kinases and BTK. In vitro studies show that dasatinib is toxic to ABC DLBCL cell lines that rely on chronic active BCR signaling, suggesting that this drug is a candidate for the treatment of DLBCL [98, 99]. Imatinib, a PDGFRA-selective tyrosine kinase inhibitor, has been validated to be effective in blocking lymphoma growth in both human xenograft and murine allograft models [100]. Although mitomycin C is not a commonly used chemotherapeutic agent for the treatment of DLBCL, the role of these TKIs as a therapeutic strategy in DLBCL has been demonstrated. Of note, the sensitivity to these chemotherapeutic agents and targeted therapies could be evaluated by our risk model. This suggests that our model may guide the personalized treatment of DLBCL patients. Subsequently, the risk score of our model was included together with other widely used prognostic indicators to construct the predictive nomogram. The risk score contributed more to survival prediction compared with the other indicators and was also confirmed as an independent prognostic factor. Therefore, our risk model could be a promising tool for clinical applications in the future.

However, there are still some limitations in our current study. Our risk model derived from bioinformatic analysis needs to be further confirmed by functional and mechanistic experiments, as only three of the genes included in the signature, TYMP, PCNA, and WRN, have been previously studied as prognostic markers for DLBCL. In addition, our current study is retrospective, so prospective studies are needed to further confirm our findings.

## 5. Conclusions

In summary, our study established a novel robust 14-gene signature based on DNA repair genes that could be used for the stratification and prognostic prediction of DLBCL patients. Our study also provided new insights into the relationship between this signature and the immune microenvironment as well as immunotherapy to guide the clinical treatment of DLBCL patients.

## Data Availability

The datasets used and/or analyzed during the current study are available upon reasonable request from the corresponding author.

## Abbreviations

DLBCL: Diffuse large B-cell lymphoma
TCGA: The Cancer Genome Atlas
GEO: Gene Expression Omnibus
GDSC: Genomics of Drug Sensitivity in Cancer
GSVA: Gene set variation analysis
ASCT: Autologous stem cell transplantation
IPI: International Prognostic Index
ROC: Receiver operating characteristic
IC50: Half-maximal inhibitory concentration
ceRNA: Competing endogenous RNA
OS: Overall survival
HR: Hazard ratio
AUC: Area under the curve
MHC: Major histocompatibility complex
AAS: Ann Arbor staging
COO: Cell of origin.

## Supplementary Materials

Supplementary 1 Table S1: 29 prognosis-related DNA repair genes were significantly predictive of prognosis in DLBCL patients by univariate Cox regression analysis (*p* < 0.001).

Supplementary 2 Table S2: top 28 genes in the PPI network.

Supplementary 3 Table S3: coefficients of 14 selected DNA repair genes.

Supplementary 4 Figure S1: the motif enrichment information and its annotation.

## References

[1] Sehn L. H., Salles G. (2021). Diffuse Large B-Cell Lymphoma. *New England Journal of Medicine*.

[2] Gisselbrecht C., Van Den Neste E. (2018). How I manage patients with relapsed/refractory diffuse large B cell lymphoma. *British Journal of Haematology*.

[3] Coiffier B., Thieblemont C., Van Den Neste E. (2010). Long-term outcome of patients in the LNH-98.5 trial, the first randomized study comparing rituximab-CHOP to standard CHOP chemotherapy in DLBCL patients: a study by the Groupe d'Etudes des Lymphomes de l’Adulte. *Blood*.

[4] Liu Y., Barta S. K. (2019). Diffuse large B-cell lymphoma: 2019 update on diagnosis, risk stratification, and treatment. *American Journal of Hematology*.

[5] (1993). A predictive model for aggressive non-Hodgkin's lymphoma. *The New England Journal of Medicine*.

[6] Johnson N. A., Slack G. W., Savage K. J. (2012). Concurrent expression of MYC and BCL2 in diffuse large B-cell lymphoma treated with rituximab plus cyclophosphamide, doxorubicin, vincristine, and prednisone. *Journal of Clinical Oncology*.

[7] Peroja P., Pedersen M., Mantere T. (2018). Mutation of TP53, translocation analysis and immunohistochemical expression of MYC, BCL-2 and BCL-6 in patients with DLBCL treated with R-CHOP. *Scientific Reports*.

[8] Young K. H., Weisenburger D. D., Dave B. J. (2007). Mutations in the DNA-binding codons of TP53, which are associated with decreased expression of TRAILreceptor-2, predict for poor survival in diffuse large B-cell lymphoma. *Blood*.

[9] Zenz T., Kreuz M., Fuge M. (2017). TP53 mutation and survival in aggressive B cell lymphoma. *International Journal of Cancer*.

[10] Juskevicius D., Jucker D., Klingbiel D., Mamot C., Dirnhofer S., Tzankov A. (2017). Mutations of CREBBP and SOCS1 are independent prognostic factors in diffuse large B cell lymphoma: mutational analysis of the SAKK 38/07 prospective clinical trial cohort. *Journal of Hematology & Oncology*.

[11] Friedman J., Hastie T., Tibshirani R. (2010). Regularization paths for generalized linear models via coordinate descent. *Journal of Statistical Software*.

[12] Heagerty P. J., Zheng Y. (2005). Survival model predictive accuracy and ROC curves. *Biometrics*.

[13] (2012). clusterProfiler: an R package for comparing biological themes among gene clusters. *OMICS: A Journal of Integrative Biology*.

[14] Geeleher P., Cox N. J., Huang R. S. (2014). Clinical drug response can be predicted using baseline gene expression levels and in vitro drug sensitivity in cell lines. *Genome Biology*.

[15] Newman A. M., Liu C. L., Green M. R. (2015). Robust enumeration of cell subsets from tissue expression profiles. *Nature Methods*.

[16] Hänzelmann S., Castelo R., Guinney J. (2013). GSVA: gene set variation analysis for microarray and RNA-Seq data. *BMC Bioinformatics*.

[17] Salmena L., Poliseno L., Tay Y., Kats L., Pandolfi P. P. (2011). A ceRNA hypothesis: the Rosetta stone of a hidden RNA language?. *Cell*.

[18] Aerts S., Quan X. J., Claeys A. (2010). Robust target gene discovery through transcriptome perturbations and genome-wide enhancer predictions in Drosophila uncovers a regulatory basis for sensory specification. *PLoS Biology*.

[19] Aibar S., González-Blas C. B., Moerman T. (2017). SCENIC: single-cell regulatory network inference and clustering. *Nature Methods*.

[20] Koh J. J., Lim S. T., Sultana R. (2018). Predictors of early vs late diffuse large B cell lymphoma (DLBCL) relapses in the rituximab era. *Journal of Clinical Oncology*.

[21] Autio M., Leivonen S. K., Brück O. (2021). Immune cell constitution in the tumor microenvironment predicts the outcome in diffuse large B-cell lymphoma. *Haematologica*.

[22] Chan A., Dogan A. (2019). Prognostic and predictive biomarkers in diffuse large B-cell lymphoma. *Surgical Pathology Clinics*.

[23] Schmitz R., Wright G. W., Huang D. W. (2018). Genetics and pathogenesis of diffuse large B-cell lymphoma. *New England Journal of Medicine*.

[24] Chapuy B., Stewart C., Dunford A. J. (2018). Molecular subtypes of diffuse large B cell lymphoma are associated with distinct pathogenic mechanisms and outcomes. *Nature Medicine*.

[25] Zhou M., Zhao H., Xu W., Bao S., Cheng L., Sun J. (2017). Discovery and validation of immune-associated long non-coding RNA biomarkers associated with clinically molecular subtype and prognosis in diffuse large B cell lymphoma. *Molecular Cancer*.

[26] Hu J., Xu J., Yu M. (2020). An integrated prognosis model of pharmacogenomic gene signature and clinical information for diffuse large B-cell lymphoma patients following CHOP-like chemotherapy. *Journal of Translational Medicine*.

[27] De Miranda N., Peng R., Georgiou K. (2013). DNA repair genes are selectively mutated in diffuse large B cell lymphomas. *The Journal of Experimental Medicine*.

[28] Ruhe M., Rabe D., Jurischka C. (2019). Molecular biomarkers of DNA damage in diffuse large-cell lymphoma—a review. *Journal of Laboratory and Precision Medicine*.

[29] Derenzini E., Agostinelli C., Imbrogno E. (2015). Constitutive activation of the DNA damage response pathway as a novel therapeutic target in diffuse large B-cell lymphoma. *Oncotarget*.

[30] Han W., Ming M., Zhao R., Pi J., Wu C., He Y.-Y. (2012). Nrf1 CNC-bZIP protein promotes cell survival and nucleotide excision repair through maintaining glutathione homeostasis^∗^. *Journal of Biological Chemistry*.

[31] Wang L., Yang B., Xu Z. (2021). NRF1-regulated CircNSUN2 promotes lymphoma progression through activating Wnt signaling pathway via stabilizing HMGA1. *Cell cycle (Georgetown, Tex)*.

[32] Johnson R. D., Liu N., Jasin M. (1999). Mammalian XRCC2 promotes the repair of DNA double-strand breaks by homologous recombination. *Nature*.

[33] Kamali M., Hamadani S., Neamatzadeh H. (2017). Association of XRCC2 rs3218536 polymorphism with susceptibility of breast and ovarian cancer: a systematic review and meta-analysis. *Asian Pacific Journal of Cancer Prevention : APJCP*.

[34] Lin W.-Y., Camp N. J., Cannon-Albright L. A. (2011). A role for XRCC2 gene polymorphisms in breast cancer risk and survival. *Journal of Medical Genetics*.

[35] Park D. J., Lesueur F., Nguyen-Dumont T. (2012). Rare mutations in XRCC2 increase the risk of breast cancer. *The American Journal of Human Genetics*.

[36] Bronckaers A., Aguado L., Negri A. (2009). Identification of aspartic acid-203 in human thymidine phosphorylase as an important residue for both catalysis and non-competitive inhibition by the small molecule "crystallization chaperone" 5′-O-tritylinosine (KIN59). *Biochemical Pharmacology*.

[37] Bronckaers A., Gago F., Balzarini J., Liekens S. (2009). The dual role of thymidine phosphorylase in cancer development and chemotherapy. *Medicinal Research Reviews*.

[38] Nie X., Clifford P. M., Bhat R., Heintzelman R., Abraham M., Hou J. S. (2013). Thymidine phosphorylase expression in B-cell lymphomas and its significance: a new prognostic marker?. *Analytical and Quantitative Cytopathology and Histopathology*.

[39] Toueille M., El-Andaloussi N., Frouin I. (2004). The human Rad9/Rad1/Hus1 damage sensor clamp interacts with DNA polymerase beta and increases its DNA substrate utilisation efficiency: implications for DNA repair. *Nucleic Acids Research*.

[40] Lopes J. L., Chaudhry S., Lopes G. S., Levin N. K., Tainsky M. A. (2019). FANCM, RAD1, CHEK1 and TP53I3 act as BRCA-like tumor suppressors and are mutated in hereditary ovarian cancer. *Cancer Genetics*.

[41] Loo S. K., Hamid S. S., Musa M., Wong K. K. (2018). DNMT1 is associated with cell cycle and DNA replication gene sets in diffuse large B-cell lymphoma. *Pathology - Research and Practice*.

[42] Xie W., Wu M., Fu T. (2017). ICT1 predicts a poor survival and correlated with cell proliferation in diffuse large B-cell lymphoma. *Gene*.

[43] Belessi C. J., Parasi A. S., Manioudaki H. S. (2003). Prognostic impact of DNA ploidy pattern, S-phase fraction (SPF), and proliferating cell nuclear antigen (PCNA) in patients with primary gastric lymphoma. *Journal of Surgical Oncology*.

[44] Pickart C. M., Fushman D. (2004). Polyubiquitin chains: polymeric protein signals. *Current Opinion in Chemical Biology*.

[45] Tang Y., Geng Y., Luo J. (2015). Downregulation of ubiquitin inhibits the proliferation and radioresistance of non-small cell lung cancer cells in vitro and in vivo. *Scientific Reports*.

[46] Scarpa E. S., Tasini F., Crinelli R., Ceccarini C., Magnani M., Bianchi M. (2020). The ubiquitin gene expression pattern and sensitivity to UBB and UBC knockdown differentiate primary 23132/87 and metastatic MKN45 gastric cancer cells. *International Journal of Molecular Sciences*.

[47] Liontos M., Koutsami M., Sideridou M. (2007). Deregulated overexpression of hCdt1 and hCdc6 promotes malignant behavior. *Cancer Research*.

[48] Tatsumi Y., Sugimoto N., Yugawa T., Narisawa-Saito M., Kiyono T., Fujita M. (2006). Deregulation of Cdt1 induces chromosomal damage without rereplication and leads to chromosomal instability. *Journal of Cell Science*.

[49] Karavias D., Maroulis I., Papadaki H. (2016). Overexpression of CDT1 is a predictor of poor survival in patients with hepatocellular carcinoma. *Journal of Gastrointestinal Surgery*.

[50] Li J.-N., Feng C.-J., Lu Y.-J. (2008). mRNA expression of the DNA replication-initiation proteins in epithelial dysplasia and squamous cell carcinoma of the tongue. *BMC Cancer*.

[51] Morgada M. N., Abriata L. A., Cefaro C., Gajda K., Banci L., Vila A. J. (2015). Loop recognition and copper-mediated disulfide reduction underpin metal site assembly of CuA in human cytochrome oxidase. *Proceedings of the National Academy of Sciences of the United States of America*.

[52] Matoba S., Kang J.-G., Patino W. D. (2006). p53 regulates mitochondrial respiration. *Science*.

[53] Won K. Y., Lim S.-J., Kim G. Y. (2012). Regulatory role of p53 in cancer metabolism via SCO2 and TIGAR in human breast cancer. *Human Pathology*.

[54] Liu J., Lu F., Gong Y. (2018). High expression of synthesis of cytochrome c oxidase 2 and TP53-induced glycolysis and apoptosis regulator can predict poor prognosis in human lung adenocarcinoma. *Human Pathology*.

[55] Albertella M. R., Lau A., O’Connor M. J. (2005). The overexpression of specialized DNA polymerases in cancer. *DNA Repair*.

[56] O-Wang J., Kawamura K., Tada Y. (2001). DNA polymerase *κ*, implicated in spontaneous and DNA damage-induced mutagenesis, is overexpressed in lung cancer. *Cancer Research*.

[57] Wang H., Wu W., Wang H.-W. (2010). Analysis of specialized DNA polymerases expression in human gliomas: association with prognostic significance. *Neuro-Oncology*.

[58] Scrima A., Konícková R., Czyzewski B. K. (2008). Structural basis of UV DNA-damage recognition by the DDB1-DDB2 complex. *Cell*.

[59] Huang S., Fantini D., Merrill B. J., Bagchi S., Guzman G., Raychaudhuri P. (2017). DDB2 is a novel regulator of Wnt signaling in colon cancer. *Cancer Research*.

[60] Han C., Zhao R., Liu X. (2014). DDB2 suppresses tumorigenicity by limiting the cancer stem cell population in ovarian cancer. *Molecular Cancer Research : MCR*.

[61] Gilson P., Drouot G., Witz A., Merlin J. L., Becuwe P., Harlé A. (2019). Emerging roles of DDB2 in cancer. *International Journal of Molecular Sciences*.

[62] Shen M., Zheng T., Lan Q. (2006). Polymorphisms in DNA repair genes and risk of non-Hodgkin lymphoma among women in Connecticut. *Human Genetics*.

[63] Grandori C., Robinson K. L., Galloway D. A., Swisshelm K. (2004). Functional link between Myc and the Werner gene in tumorigenesis. *Cell Cycle (Georgetown, Tex)*.

[64] Qu C., Zhao Y., Feng G. (2017). RPA3 is a potential marker of prognosis and radioresistance for nasopharyngeal carcinoma. *Journal of Cellular and Molecular Medicine*.

[65] Dai Z., Wang S., Zhang W., Yang Y. (2017). Elevated expression of RPA3 is involved in gastric cancer tumorigenesis and associated with poor patient survival. *Digestive Diseases and Sciences*.

[66] Xiao W., Zheng J., Zhou B., Pan L. (2017). Replication protein A 3 is associated with hepatocellular carcinoma tumorigenesis and poor patient survival. *Digestive diseases (Basel, Switzerland)*.

[67] Kobayashi K., Guilliam T. A., Tsuda M. (2016). Repriming by PrimPol is critical for DNA replication restart downstream of lesions and chain-terminating nucleosides. *Cell cycle (Georgetown, Tex)*.

[68] Tirman S., Cybulla E., Quinet A., Meroni A., Vindigni A. (2021). PRIMPOL ready, set, reprime!. *Critical Reviews in Biochemistry and Molecular Biology*.

[69] González-Acosta D., Blanco-Romero E., Mutreja K. (2021). PrimPol primase mediates replication traverse of DNA interstrand crosslinks. *bioRxiv*.

[70] Watkins J. F., Sung P., Prakash L., Prakash S. (1993). The Saccharomyces cerevisiae DNA repair gene RAD23 encodes a nuclear protein containing a ubiquitin-like domain required for biological function. *Molecular and Cellular Biology*.

[71] Kaur M., Pop M., Shi D., Brignone C., Grossman S. R. (2007). hHR23B is required for genotoxic-specific activation of p53 and apoptosis. *Oncogene*.

[72] Kolodner R. D., Marsischky G. T. (1999). Eukaryotic DNA mismatch repair. *Current Opinion in Genetics & Development*.

[73] Simon J. A., Szankasi P., Nguyen D. K. (2000). Differential toxicities of anticancer agents among DNA repair and checkpoint mutants of Saccharomyces cerevisiae. *Cancer Research*.

[74] De’ Angelis G. L., Bottarelli L., Azzoni C. (2018). Microsatellite instability in colorectal cancer. *Acta bio-medica : Atenei Parmensis*.

[75] Liu Y., Zhou X., Wang X. (2021). Targeting the tumor microenvironment in B-cell lymphoma: challenges and opportunities. *Journal of Hematology & Oncology*.

[76] Wang J., Ke X. Y. (2011). The four types of Tregs in malignant lymphomas. *Journal of Hematology & Oncology*.

[77] Serag El-Dien M. M., Abdou A. G., Asaad N. Y., Abd El-Wahed M. M., Kora M. (2017). Intratumoral FOXP3+ regulatory T cells in diffuse large B-cell lymphoma. *Applied Immunohistochemistry & Molecular Morphology: AIMM*.

[78] Tzankov A., Meier C., Hirschmann P., Went P., Pileri S. A., Dirnhofer S. (2008). Correlation of high numbers of intratumoral FOXP3+ regulatory T cells with improved survival in germinal center-like diffuse large B-cell lymphoma, follicular lymphoma and classical Hodgkin's lymphoma. *Haematologica*.

[79] Fisch P., Malkovsky M., Braakman E. (1990). Gamma/delta T cell clones and natural killer cell clones mediate distinct patterns of non-major histocompatibility complex-restricted cytolysis. *The Journal of Experimental Medicine*.

[80] Ma C., Zhang Q., Ye J. (2012). Tumor-infiltrating *γδ* T lymphocytes predict clinical outcome in human breast cancer. *Journal of immunology (Baltimore, Md: 1950)*.

[81] Patil R. S., Shah S. U., Shrikhande S. V., Goel M., Dikshit R. P., Chiplunkar S. V. (2016). IL17 producing *γδ*T cells induce angiogenesis and are associated with poor survival in gallbladder cancer patients. *International Journal of Cancer*.

[82] Park J. H., Lee H. K. (2021). Function of *γδ* T cells in tumor immunology and their application to cancer therapy. *Experimental & Molecular Medicine*.

[83] Reboursiere E., Gac A. C., Garnier A. (2018). Increased frequencies of circulating and tumor-resident V*δ*1(+) T cells in patients with diffuse large B-cell lymphoma. *Leukemia & Lymphoma*.

[84] Charwudzi A., Meng Y., Hu L. (2021). Integrated bioinformatics analysis reveals dynamic candidate genes and signaling pathways involved in the progression and prognosis of diffuse large B-cell lymphoma. *PeerJ*.

[85] Josefsson S. E., Beiske K., Blaker Y. N. (2019). TIGIT and PD-1 mark intratumoral T cells with reduced effector function in B-cell non-Hodgkin lymphoma. *Cancer Immunology Research*.

[86] Jung M., Russell A. J., Liu B. (2017). A Myc activity signature predicts poor clinical outcomes in Myc-associated cancers. *Cancer Research*.

[87] Valera A., López-Guillermo A., Cardesa-Salzmann T. (2013). MYC protein expression and genetic alterations have prognostic impact in patients with diffuse large B-cell lymphoma treated with immunochemotherapy. *Haematologica*.

[88] Karlsson A., Deb-Basu D., Cherry A., Turner S., Ford J., Felsher D. W. (2003). Defective double-strand DNA break repair and chromosomal translocations by MYC overexpression. *Proceedings of the National Academy of Sciences*.

[89] Felsher D. W., Bishop J. M. (1999). Transient excess of MYC activity can elicit genomic instability and tumorigenesis. *Proceedings of the National Academy of Sciences of the United States of America*.

[90] Williams A. B., Schumacher B. (2016). p53 in the DNA-damage-repair process. *Cold Spring Harbor Perspectives in Medicine*.

[91] Hetz C., Zhang K., Kaufman R. J. (2020). Mechanisms, regulation and functions of the unfolded protein response. *Nature Reviews Molecular Cell Biology*.

[92] González-Quiroz M., Blondel A., Sagredo A., Hetz C., Chevet E., Pedeux R. (2020). When endoplasmic reticulum proteostasis meets the DNA damage response. *Trends in Cell Biology*.

[93] Bolland H., Ma T. S., Ramlee S., Ramadan K., Hammond E. M. (2021). Links between the unfolded protein response and the DNA damage response in hypoxia: a systematic review. *Biochemical Society Transactions*.

[94] Zhang Y., Wang X. (2020). Targeting the Wnt/*β*-catenin signaling pathway in cancer. *Journal of Hematology & Oncology*.

[95] Waters A. M., Der C. J. (2018). KRAS: The critical driver and therapeutic target for pancreatic cancer. *Cold Spring Harbor Perspectives in Medicine*.

[96] Karimaian A., Majidinia M., Bannazadeh Baghi H., Yousefi B. (2017). The crosstalk between Wnt/*β*-catenin signaling pathway with DNA damage response and oxidative stress: implications in cancer therapy. *DNA Repair (Amst)*.

[97] Ludovini V., Ricciuti B., Tofanetti F. R. (2018). KRAS mutation and DNA repair and synthesis genes in non-small-cell lung cancer. *Molecular and Clinical Oncology*.

[98] Hantschel O., Rix U., Schmidt U. (2007). The Btk tyrosine kinase is a major target of the Bcr-Abl inhibitor dasatinib. *Proceedings of the National Academy of Sciences*.

[99] Davis R. E., Ngo V. N., Lenz G. (2010). Chronic active B-cell-receptor signalling in diffuse large B-cell lymphoma. *Nature*.

[100] Ruan J., Luo M., Wang C. (2013). Imatinib disrupts lymphoma angiogenesis by targeting vascular pericytes. *Blood*.

