# Supplementary material for: Identification and Validation of a DNA Damage Repair-Related Signature for Diffuse Large B-Cell Lymphoma
Source: Biomed Res Int. 2022 Oct 14;2022:2645090. doi: 10.1155/2022/2645090 (PMC9587677; doi:10.1155/2022/2645090)
Supplement: Supplementary 4 — Figure S1: the motif enrichment information and its annotation. [file 2645090.f4.pdf]

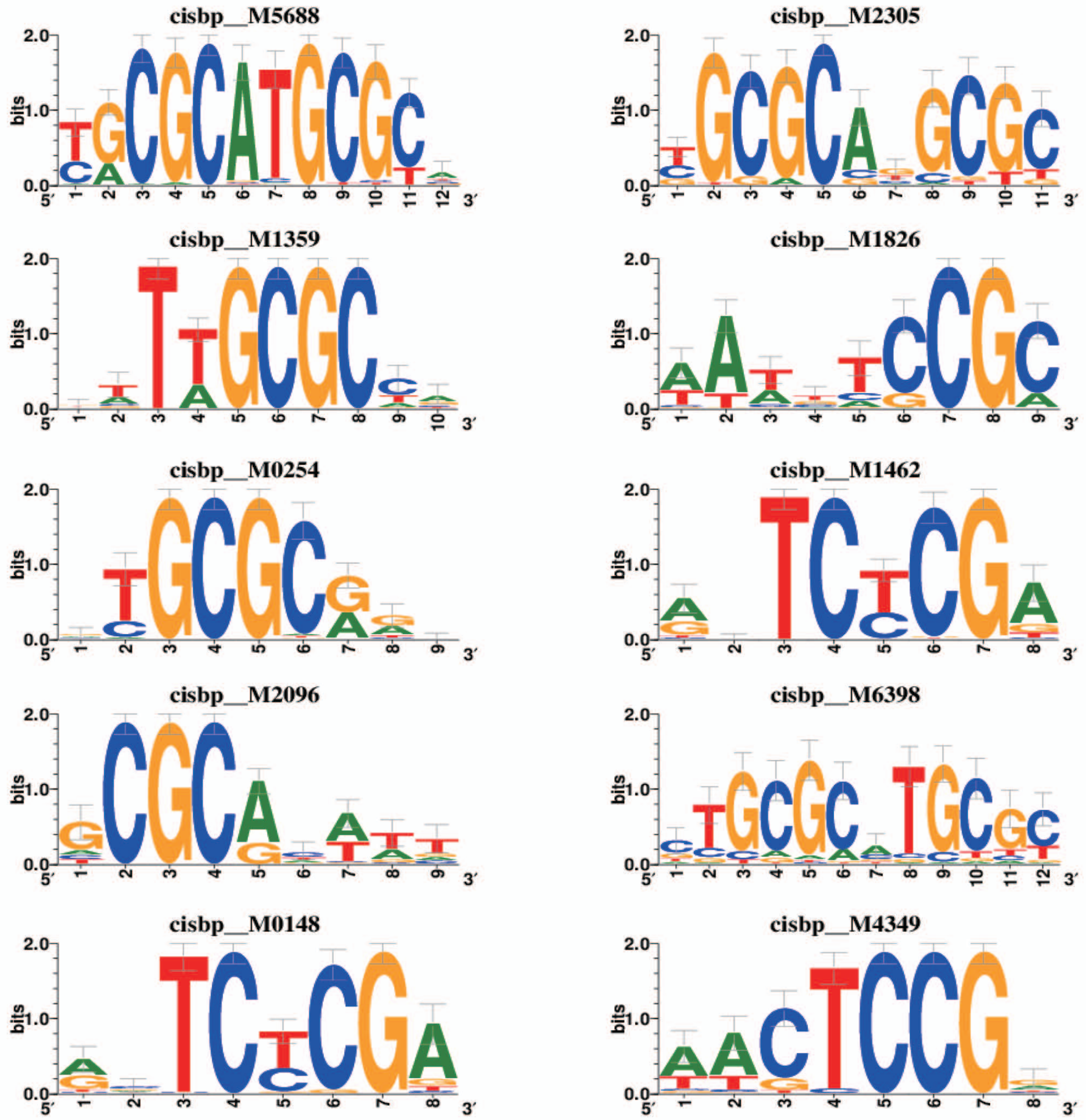

	logo	geneSet	motif	NES	AUC	TF_highConf	nEnrGenes	rankAtMax
	All	All	All	All	All	All	All	All
1		lasso_gene	cisbp__M5688	9.75	0.243	NRF1 (directAnnotation).	5	867
2		lasso_gene	cisbp__M2305	8.6	0.216	NRF1 (directAnnotation).	5	867
3		lasso_gene	cisbp__M1359	7.71	0.194	MYPOP (inferredBy_Orthology).	4	1503
4		lasso_gene	cisbp__M1826	7.7	0.194		4	867
5		lasso_gene	cisbp__M0254	7.57	0.191		6	3429

	logo	geneSet	motif	NES	AUC	TF_highConf	nEnrGenes	rankAtMax
	All	All	All	All	All	All	All	All
6		lasso_gene	cisbp__M1462	7.27	0.184		3	470
7		lasso_gene	cisbp__M2096	7.25	0.183		4	1503
8		lasso_gene	cisbp__M6398	6.66	0.169	NRF1 (directAnnotation).	5	1503
9		lasso_gene	cisbp__M0148	6.54	0.166		3	867
10		lasso_gene	cisbp__M4349	5.64	0.145		3	1503
